# “The bottom line is that it is all about trust”: Interviews with Health Services Administrators about perceived barriers and facilitators to vaccine administration in jails

**DOI:** 10.1017/cts.2022.519

**Published:** 2023-01-23

**Authors:** Nicole Cassarino, Laura Lodolo, Emma Smyth, Megha Ramaswamy, Alysse Wurcel

**Affiliations:** 1 Tufts University School of Medicine, Boston, MA, USA; 2 Department of Medicine, Division of Geographic Medicine, and Infectious Diseases, Tufts Medical Center, Boston, MA , USA; 3 Department of Population Health, University of Kansas School of Medicine, Kansas City, KS, USA

**Keywords:** Vaccine hesitancy, COVID-19, jail, incarceration, Health Services Administrators

## Abstract

**Background:**

Compared to the general population, individuals incarcerated in jails and prisons are more vulnerable to infection and mortality from communicable diseases, such as COVID-19 and influenza. However, vaccination rates among incarcerated individuals as well as staff who work in jails and prisons remain disproportionately low. Healthcare administrators working in jails have first-hand experience about barriers to vaccine provision, but their perspectives are infrequently collected and analyzed.

**Methods:**

We reached out to Health Services Administrators (HSAs) from all 14 Massachusetts (MA) county jails for qualitative in-depth interviews to understand how their personal and professional feelings about vaccination relate to the barriers and facilitators that surround administration of vaccines in jail.

**Results:**

Eight people participated in the study (8/14 = 57% response rate). Key themes emerged, including 1) HSAs expressed divergent opinions on incarceration as the correct opportunity to vaccinate individuals, 2) HSAs’ personal views on vaccines influenced their operationalization of vaccination in jail, and 3) opinions varied on whether their institutions’ vaccine protocols needed modification.

**Conclusions:**

Our findings highlight the critical need to leverage the feedback and influence of stakeholders such as HSAs in efforts to improve preventative healthcare delivery in carceral health systems.

## Introduction

Vaccination is a crucial strategy for mitigating the spread of infectious diseases in conjugate settings such as jails and prisons. In 2020, case rates of COVID-19 among individuals incarcerated in the USA jails and prisons exceeded those observed in the general, noninstitutionalized population by a factor of 5.5 [1]. The current COVID-19 pandemic is part of a trend of high rates of infectious diseases in correctional settings that has lasted for decades; for example, in 2011–2012, HIV rates were three times higher in US jails and prisons than in the general population [2]. Additionally, the amount of older individuals who are incarcerated has increased over recent years, indicating the increased susceptibility to infectious disease-related mortality experienced by many justice-involved individuals [3].

Despite increased risk for infections, studies show low vaccination rates in justice-involved populations and people who work in jails and prisons. For example, as of October 2021, approximately 60% of Massachusetts corrections workers are fully vaccinated against COVID-19, compared to 70.5% of all US adults [4,5]. Similarly, previous work from our lab found that, on average, less than 12% of individuals incarcerated in MA jails were vaccinated against influenza between 2013 and 2019, much lower than the average influenza vaccination rate of 30–50% among US adults during that period [6,7]. Vaccine nonintent, also known as vaccine hesitancy, is often cited as one of the most substantial barriers to vaccinating people who are incarcerated [8,9,10].

Most of the research to date on improving vaccine delivery in jails has focused on education, engagement, and building trust with people who are incarcerated [11,12]. Vaccine nonintent in people who are incarcerated is not the only barrier, however. The personal views on vaccination of people who work in jail, especially the people who oversee healthcare, may influence if and how vaccines are operationalized in jails. Previous work completed on implementation of HPV vaccination in jails found that stakeholder investment with jail administrators was crucial to improving vaccination rates [13]. Studies have also found that, historically, building relationships between stakeholders working in jails, including sheriffs and nurses, and local health departments leads to better management of infectious diseases, including HCV and influenza [14,15].

We interviewed Health Services Administrators (HSAs) in MA county jails on their personal and professional views of vaccination to further understand barriers and facilitators to vaccination in the jails. The health services administrator is a person in Massachusetts jails is the person who oversees all medical and administrative tasks in the jails. This person can be employed by the county, or can be employed by the contracted health organization. People in this position are often nurses but this is not required. Jails often have a separate person who is the director of nursing.

It is important to highlight that our investigation focused on the perspectives of people working in jails, not prisons. Prisons house individuals post-trial, generally for a specified sentence, whereas jails are the entry point of the US correctional system, housing arrested individuals pretrial and pre-sentence for much shorter periods of time, on average. Research has shown that rates of infectious disease in surrounding, nonincarcerated communities cannot be decreased until public health efforts turn their focus toward local jails [16]. Understanding the beliefs and attitudes around vaccination held by HSAs, the people who provide health services to individuals incarcerated in jails, serves as a crucial first step in identifying and implementing the changes needed to improve vaccine uptake rates in MA county jails.

## Materials and Methods

Invitations to participate in interviews were sent via e-mail to Health Services Administrators in each of the 14 Massachusetts county jails between February 24, 2021 and March 18, 2021. Interviewees and the interviewer did not meet before their interview; the only prior communication included a brief introduction about the interviewer, her involvement in the research as a medical student studying carceral health and the purpose of the study. All interviews were conducted one-on-one with only the HSA participant and interviewer, (N.C.) who is a female medical student and MPH graduate, present on Zoom® teleconferencing software. The interviewer had completed a Master’s level course in qualitative research methods. Interviews took place between 2/24/21 and 8/17/21. Each participant was interviewed only once. Interviews were not recorded; the interviewer transcribed interviews in real-time and recorded field notes. Participants did not see their interview transcripts at any time. Each interview lasted between 0.5 and 1.0 hours. All participants were deidentified, and no data was asked of any participant because of the small number of participants and the potential that any demographic information could make the participants’ responses identifiable. Respondents were not compensated for their time.

We designed the qualitative interview guide based on the Theoretical Domain Framework (TDF), an integrative conceptual model created to study roots of behavior change across multiple research disciplines [17]. While a variety of models and frameworks could have been chosen, this model was used because of its utility in probing the underlying determinants of behavior when implementing new practices, which might provide insights from stakeholders that could be operationalized to improve practices around vaccine administration in jail. Furthermore, use of the TDF has supported the development of techniques that result in evidence-based behavior change, particularly at the individual level [18,19]. Using this framework allowed us to propose evidence-based strategies for behavior change among HSAs that could increase vaccination rates of both staff and incarcerated individuals in MA jails. Although factors beyond the individual level (i.e., institutional, political, etc.) contribute to a person’s attitudes and behaviors around vaccination, we utilized the TDF because it aligned with our study design, which focused on the individual’s role in their own behavior change.

The model contains twelve domains in its original version and fourteen in its newer validated version. Because we aimed to capture a holistic view of HSAs’ behavior around vaccine administration in jails, we used the newer TDF domains and all fourteen domains of the TDF were included in our interview guide. These included (1) knowledge; (2) skills; (3) social or professional role and identity; (4) beliefs about capabilities; (5) optimism; (6) beliefs about consequences; (7) reinforcement; (8) intentions; (9) goals; (10) memory, attention, and decision processes; (11) environmental context; (12) social influences; (13) emotion; and (14) behavioral regulation. Each domain included multiple theoretical constructs related to behavior change, captured by the fourteen overarching theoretical domains. Supplement 1 in the Supplementary Materials includes a list of all of the domains of the TDF and a description of how interview guide questions were created to reflect these fourteen domains and their constructs. Given the small sample size to which we had access, HSAs were not available to pilot the instrument.

Interview questions were not specific to any particular vaccine; rather interviewees were asked about attitudes surrounding vaccination in general. Interviewees did not see the interview guide prior to the interview session. The transcripts were coded by three co-authors (N.C., L.L., and E.S.) who iteratively discussed codes until a consensus was reached for each transcript. The codes used were a combination of 1) factors that contribute to vaccine hesitancy as developed by the SAGE Working Group on Vaccine Hesitancy and 2) codes created by the authors as deemed appropriate and useful through an inductive coding approach [20]. The interview guide and codebook are available in the Supplementary Materials (Supplements 2 and 3, respectively). Qualitative analyses of the interview transcripts were conducted iteratively using Dedoose software (v9.0.17). Tufts Health Sciences IRB approved this study.

## Results

Of the fourteen HSAs invited, a total of eight HSAs/institutions agreed to participate in interviews. The six invited HSAs who did not participate did not respond to the initial and follow-up invitiations. Respondents included HSAs from two small-medium jails (capacity of 51–249 persons) and 6 large-mega jails (capacity of 250 to over 1000 persons)[21]. No demographic data was collected from the interviewees to maintain their privacy, given the small sample size and potential for identification. Participants were not asked for feedback on the findings.

**Fig. 1.**
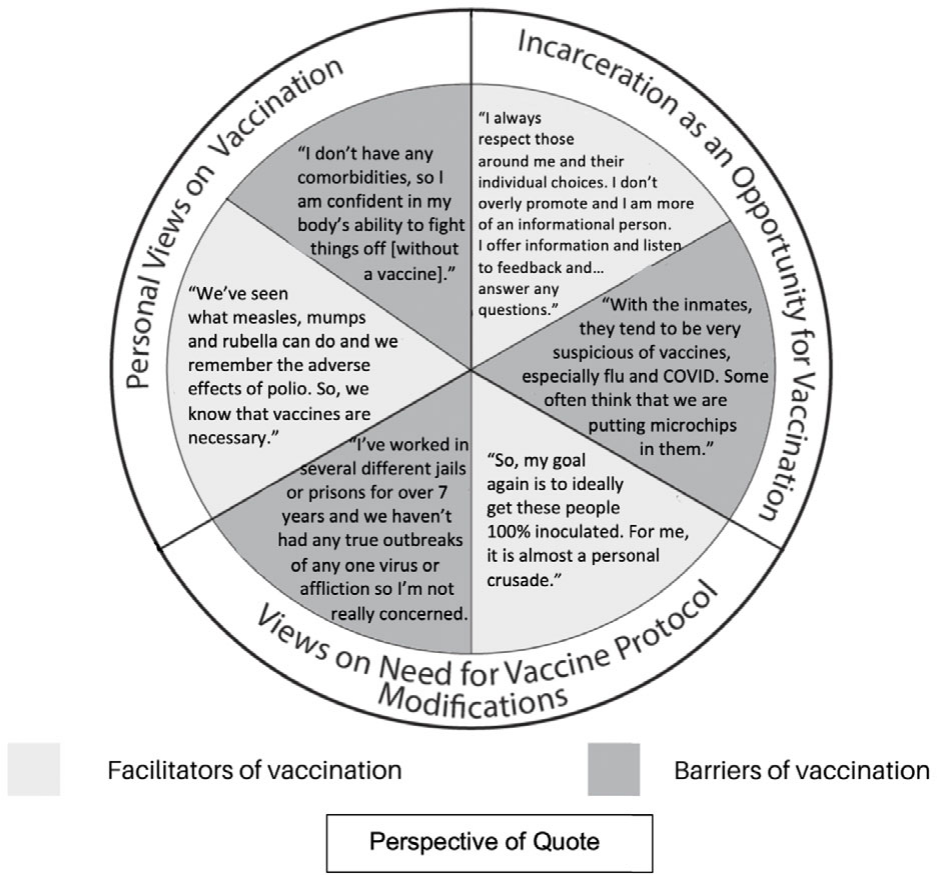
Selected quotes from Health Services Administrators interviews reflecting three key emergent themes.

While consensus coding interview transcripts, the authors recognized overarching themes that were derived from the data and ran through each of the eight interviews. A total of three key themes emerged: (1) incarceration as an opportunity for vaccination, (2) personal views on vaccination influencing operationalization, and (3) views on the necessity for jail vaccine protocol modifications. These three themes were created by identifying patterns that ran through coded transcripts. Collectively, these themes encapsulate all twenty-five codes, some of which were included in more than one theme when appropriate, as seen in Supplement 4 in the Supplementary Materials. The number of interviewees (out of a maximum of *N* = 8) and the total number of times the code was applied to an interview transript are listed in Supplement 4 as well. Figure 1 and Table 1 list facilitators and barriers associated with each theme. It is worth noting that due to the limited sample of HSAs sampled, we were unable to assess theme saturation, though reports of how many unique interviewees referenced any given theme are recorded in Supplement 4.

**Table 1.** Barriers and facilitators associated with each theme

Theme	Barriers	Facilitators
Personal views on vaccination	• Views vaccination as personally unnecessary (i.e. due to confidence in immune system)	• History and past experience with infectious diseases promote interest in vaccination
Incarceration as an opportunity for vaccination	• Some incarcerated individuals are suspicious of correctional healthcare • High turnover complicates vaccine delivery	• During incarceration, healthcare staff are easily accessible to discuss vaccination concerns, building trust • No cost for vaccination (in Massachusetts jails)
Views on need for vaccine protocol modifications	• Lack of institutional history of infection outbreaks makes vaccine protocol modification seem unnecessary • Benchmarking against other jails with lower vaccination rates suggests no need to increase rates	• Aspiration to vaccinate all incarcerated individuals motivates the need to update vaccine protocols in jail

### Incarceration as an Opportunity for Vaccination

There were mixed views about whether incarceration was the correct opportunity for vaccination. Some HSAs acknowledged the hardships of administering vaccines in jail, including high rates of turnover, distrust of the vaccine and/or the healthcare system and the need to respect the personal choice of some individuals to not get vaccinated. Some HSAs felt that high rates of turnover in their jails complicated ordering, storage, and administration of vaccines. One HSA cited difficulties associated with obtaining a history of vaccination from incarcerated individuals, explaining: “They can be poor historians in that they don't know if they’ve had it before or not. It’s hard to get their records if they say they have received it so we send the request but we don't always get the info back that we want or need” (Participant 4).

Several HSAs also felt that peer pressure and the media fueled misinformation and distrust more for people who are incarcerated than for those living in the community. One HSA said, “With the inmates, they tend to be very suspicious of vaccines, especially flu and COVID. Some often think that we are putting microchips in them.” (Participant 10) When asked to name some barriers to vaccination of their incarcerated individuals, several HSAs discussed the challenges of fostering trust with incarcerated individuals about vaccines when many of them did not have trust in the correctional healthcare system or the healthcare system in general. One HSA said a major barrier was “overcoming stereotypes and superstitions. A lot of those are just based on prior encounters or based on cultural norms where individuals make those decisions based on their culture” (Participant 1).

Alternatively, some HSAs cited factors that support the notion of jail as the correct place to administer vaccines. Vaccines in jails, at least in MA, are administered free of charge, and several HSAs felt free vaccines were a facilitator toward vaccination during incarceration. Several discussed how incarceration promoted the ability to use their clinical training and proximity to the incarcerated population to build trust. One HSA said, "The bottom line is that it is about trust. If I can demonstrate consistency and reliability in answering their questions and care for them and develop a bond and a trustful relationship, then we can get these individuals inoculated because they will have faith in us.” (Participant 3) Another HSA said, “Most people want information. Most people want me to tell them if they should take it or not…they have a lot of questions.” (Participant 2) Another mentioned, “I always respect those around me and their individual choices. I don’t overly promote and I am more of an informational person; I offer information and listen to feedback and do what I can to answer any questions and I hope for the best. We never want to step on anyone’s toes so we do our best to ensure that everyone feels that their voice is heard and that their choice matters.” (Participant 6) From our interviews, we discovered that the lack of cost associated with the vaccine, the trust which some incarcerated individuals place in HSAs and the information which HSAs can share with incarcerated individuals can all be leveraged to promote higher rates of vaccination among populations living in jails.

### Personal Views on Vaccination

Some HSAs did not believe in vaccination as a necessary practice. One HSA explained their reasoning, saying,”It’s not an emotion. It’s the fact that I am healthy and I’ve been healthy without having cold, fever, chills, flu for over 35 years and this is without the vaccines.” (Participant 3) One person said, “I don't want to say I’m self-centered but it’s my decision, my knowledge. I’m not a follower. I guess the COVID vaccine is the best example, but I read the research out there. I do my own investigating. I make my own conclusions and go from there.” (Participant 8) When probed for information about this “research” the interviewee declined to elaborate. Another HSA said, “I don't have any comorbidities so I am confident in my body’s ability to fight things off [without a vaccine]” (Participant 6). Similar to respect for “personal choice” of people who are incarcerated, one HSA highlighted the role of personal choice for healthcare workers, “For the healthcare workers, its about personal preference: how they feel in their comfort zone, what they want to subject their bodies to… We’re all trained professional medical personnel with very similar backgrounds so it comes down to their personal preferences.” (Participant 1) Another HSA commented on some staff’s attitudes toward opting into vaccination, saying “I think that some people just don't want them. Especially if they are healthy and they think that they won't get the flu or COVID-19” (Participant 8). One HSA reported discussing concerns about vaccines with other staff, “Sometimes they ask, ‘Hey, are you going to get the vaccines?’ I personally say ‘no, there is not enough info out there, it’s not FDA approved, the science doesn't line up for me’” (Participant 4).

Other HSAs were supportive of vaccines, citing positive personal experiences. One HSA expressed their support while referencing their own experiences, saying “I had never had the flu before. But last year, I got vaccinated against it and I was so sick…I know that I had the flu after that vaccine. But if I hadn't gotten the vaccine, would I have been more sick? So I was grateful that I was only sick for four days, rather than longer” (Participant 1). Another HSA spoke about their support for vaccination in jail based on historical examples of infectious disease outbreaks, saying, “We’ve seen what measles, mumps, rubella can do and we remember the adverse effects and polio. So we know that vaccines are necessary” (Participant 3). One person said, “My goal again is ideally to get these people 100% inoculated. For me, it is almost a personal crusade and I would like to see as many as possible inoculated, so we can all feel a little better. It’s a huge priority for me” (Participant 8). One HSA said, “I wish there was more that we could do to make it mandatory possibly. Unfortunately, we can't do that. Not only with staff but also the inmates…they have their rights and their decisions are respected. We can only encourage them to the best of our ability” (Participant 7).

Respecting the “personal choice” of incarcerated individuals, including those who maintained a hesitant attitude toward vaccination, was particularly important during vaccine operationalization. One HSA alluded to balancing the desire to respect incarcerated individuals’ autonomy while preserving their safety saying, “Ensuring the wellbeing of folks…being able to relax based on being vaccinated. Not using those as a way to have folks change their mind because of those things, not a carrot dangling…but it’s more of a personal choice, based on the preservation of life” (Participant 4). One HSA referenced educators from the community as being incredibly important, potentially even more important than the jail healthcare staff, saying, “They don’t just hear from staff but also from people in the community who really care about them and their health and wellness” (Participant 5). Other HSAs felt that the individual’s own knowledge and experiences would inform their decisions, as one said: “The inmates understand the benefits. For example, a guy who worked in construction knows that he should get the tetanus shot” (Participant 1). Thus, through conversations with MA jail HSAs, we uncovered that the barriers of high transience, low trust, and respect for personal choice will all need to be considered in future initiatives to increase vaccination rates among individuals incarcerated in jail.

### Views on Need for Jail Vaccine Protocol Modifications

Several HSAs felt that their current institutional vaccination practices were adequate, and nothing should be changed. When asked how important it was to improve vaccination rates in their institution, one HSA responded, “To me personally? Not very. Overall, I’ve worked in several different jails or prisons for over seven years, and we haven’t had any true outbreaks of any one virus or affliction, so I’m not really concerned” (Participant 6). When asked how they felt about their jail’s vaccination rates compared to others, some expressed confidence that their rates were adequate. One HSA explained: “We look at the numbers and at this point our average percentage of people vaccinated is 52%. So we are above average at our facility, because the state average is around 35%” (Participant 4). Some felt that improvement was necessary, including one who shared, “So my goal again is ideally to get these people one hundred percent inoculated. For me it is almost a personal crusade, and I would like to see as many as possible inoculated so we can all feel a little better. It’s a huge priority for me” (Participant 1). In several interviews, benchmarking, or comparing the number of people they have vaccinated to their personal goal or other institutions, was a concept that HSAs used to justify for change in policies, or defend against the need for change.

## Discussion

Decarceration remains one of the most important public health tenets, but second to decarceration, there needs to be a focus on improving access to healthcare for people who are incarcerated. Most public health experts agree that incarceration is an optimal time to offer vaccination [22]. One action we suggest is for jails to consider quality improvement projects aimed at reviewing and improving current vaccine delivery algorithms and operations. Quality improvement studies are encouraged by several national groups, including the National Commission on Correctional Health Care [23–26]. Previous work from our lab conducted before the COVID-19 pandemic found that the percentage of individuals in MA jails who were vaccinated against influenza ranged from 1.9% to 11.8%, signifying that even the most successful jails still had less than half of their incarcerated population unvaccinated [6]. The involvement of HSAs in the evaluation and improvement of their facility’s vaccination program is critical. A study of HPV vaccination in midwestern jails found that the involvement of sheriffs alone was not enough to improve vaccination rates; those jails at which nurses coordinated and administered vaccinations managed their campaigns better with local health departments than those which did not [13].

As a first step toward quality improvement for vaccine delivery in carceral settings, we recommend appointing a “vaccine champion” to integrate the evaluation and tracking of healthcare operations into existing carceral healthcare systems. Practically, they could do this by regularly holding check-ins with the facility’s healthcare team to consistently share tracked information, such as the number of vaccines administered each month, and asking for suggestions from HSAs on how to improve these numbers. A study of 1338 nursing homes found that those which had the highest rates of staff vaccination coverage against COVID-19 were those with designated frontline vaccine champions on staff [27]. The champion could review the processes of ordering, storing, and administering vaccines. For example, the high rates of turnover often cited as a barrier to vaccination of individuals in jail could be mitigated by having a vaccine champion responsible for reviewing vaccine requests and ensuring they are completed within a pre-set amount of time. Another barrier of concern included incarcerated populations not remembering their vaccine histories. The champion could be responsible for working with state and national vaccine tracking systems to help pull vaccine data into the jail electronic medical record.

Jails should have a healthcare work force who can provide consistent, reliable, scientifically based healthcare recommendations. Some HSAs expressed concerns about vaccines. Notably, these interviews were done in spring and summer 2021, when the vaccines for COVID-19 were relatively new. Some of the HSAs may have decided to get the vaccine for COVID-19 after those dates as there was increasing data about safety. Vaccine hesitancy among correctional healthcare staff is particularly problematic as vaccine hesitant individuals have significantly less trust in physicians [28]. Until now, efforts to increase vaccination among hesitant populations, including the incarcerated, have focused mainly on education and building trust. However, education has its limits, as some people remain wary of vaccines, even with information, and respond better to incentives, such as entry into a statewide lottery, than education [29]. Jails should consider increased incentives to promote vaccination for all employees, and especially for jail employees who are working in healthcare.

Individuals incarcerated in jails have higher than average rates of infectious diseases compared to the general population, and cycle between jail and the community much faster than individuals incarcerated in prison; in 2016, around 600,000 individuals in the USA were released from federal and state prisons, while over 9 million cycled through local jails [30]. Given the differences in jails and prisons, we anticipate that barriers and facilitators of vaccination in jails might not apply to the prison setting. As mentioned previously, rates of turnover in jails far exceed those of prisons, creating shorter windows for healthcare interventions in jail and less opportunities for continuous care. Additionally, jails house the newly arrested, usually for less than one year, whereas prisons hold those serving longer sentences. As such, jails may face the barrier of not having an individual’s medical records upon admission, whereas state prison systems generally have longer periods of time to acquire medical records and establish continuous healthcare for incarcerated individuals [31]. One potential facilitator of vaccination in jails compared to prisons is that the medical intake process is shorter in jail, and people can be arrested in different counties over time, creating more opportunities to interface with healthcare in jails upon intake than there are in prisons, each of which is an opportunity to be offered vaccination.

We are incredibly grateful that eight HSAs took time from their schedules and trusted the process of research enough to contribute to our study. Still, barriers such as union-related stipulations, limited time, and questions about whether paid participation in research are permitted by employers prevent people who work in jails from participating in research. Despite the key role that HSAs play, the voices of HSAs and other administrators have often been underrepresented in research and policy formation, despite increasing research published on the importance of analyzing perspectives of criminal justice professionals when developing improved policies and protocols. Several national organizations, including the National Commission on Correctional Health Care, encourage quality improvement (QI) projects. Given the unique value of their insights as uncovered through this research, HSAs and other corrections administrators should be invited to attend academic research meetings, such as the American Consortium on Criminal Justice Health, to better understand how research and QI projects can be conducted ethically. As several national jail healthcare credentialing agencies require QI projects, HSAs should be encouraged and even paid to take coursework on QI methodology and apply to projects in jails.

In conducting this research, we encountered the following limitations. Foremost, given social-distancing requirements necessitated by the ongoing COVID-19 pandemic, we were unable to conduct interviews in person. Second, we recognize that our results might not be entirely generalizable to jail systems outside of Massachusetts. Given that MA tends to be a less vaccine-hesitant state and that vaccines are administered free of charge to individuals incarcerated in MA, our results might not apply to states where these conditions do not hold (P. Talebian, MA, MPH, e-mail communication, June 26, 2020). Relatedly, the jails represented in this study vary widely in size, as do the nearly 3000 jails in the USA [32]. We recognize that jails with small populations (i.e., those with 10 beds) will face different logistical challenges in vaccine ordering and administration than those that are larger (i.e., those with thousands of beds). We also recognize that not all of the jail healthcare administration in the USA is handled by HSAs; one of the most challenging aspects of research in jail clinical structure is the variability nationally of systems of healthcare administration. The lessons we learned from HSAs are important, but the impact may not be generalizable to other states that use different systems of healthcare administration.

Additionally, we acknowledge the potential for social desirability bias in our findings as the principal investigator of this study works closely with HSAs to administer infectious disease care in the MA jail system. However, despite this relationship, several HSAs felt comfortable enough to describe their personal distrust of vaccines, suggesting that we were able to get a variety of genuine responses. We conducted these interviews in summer of 2021, and since that time there has been more research on the safety of vaccines and increased uptake. As a result, the views of the HSAs presented here may have changed. Finally, our sample size of 8 HSAs poses a limitation; although this represents over 50% of the 14 total MA HSAs, we recommend interpreting these findings cautiously.

## Conclusions

To our knowledge, this is one of the first studies to survey Health Services Administrators about their beliefs and attitudes of vaccine delivery in jails. Given the spectrum of attitudes toward vaccination among the administrators surveyed, it is crucial that these key stakeholders are understood and engaged prior to implementation of any procedures to ultimately improve vaccination rates in jails.

## Data Availability

Not applicable.

## Supplementary material

For supplementary material accompanying this paper visit https://doi.org/10.1017/cts.2022.519. click here to view supplementary material
