# Supplementary material for: “The bottom line is that it is all about trust”: Interviews with Health Services Administrators about perceived barriers and facilitators to vaccine administration in jails
Source: J Clin Transl Sci. 2023 Jan 23;7(1):e50. doi: 10.1017/cts.2022.519 (PMC10052395; doi:10.1017/cts.2022.519)
Supplement: Supplementary file 1 [file S2059866122005192sup001.docx]

**Supplementary Materials:**

*Supplement 1. Domains and constructs of the Theoretical Domain Framework, adapted for the interview guide*

| **Domain*** | **Constructs*** | **Interview Question** |
| --- | --- | --- |
| Knowledge  (an awareness of the existence of something) | Knowledge (including knowledge of condition/scientific rationale)  Procedural knowledge  Knowledge or task environment | What types of vaccines do you give in jail?  PROBE: are any of these vaccines mandatory?  How do you think your jails does compared to other jails with getting people vaccinated? |
| Behavioral regulation (anything aimed at managing or changing objectively observed or measured actions) | Action planning  Self-monitoring  Breaking habit | Tell me about the process of giving someone a vaccine in jail?  PROBE: Is this process different depending on the type of vaccine (i.e. influenza, Hepatitis A, etc)?  How was this process developed?  PROBE: Do you know if any guidelines were used to develop this process? |
| Memory, attention and decision processes  (The ability to retain information, focus selectively on aspects of the environment and choose between two or more alternatives) | Memory  Attention  Attention control  Decision making  Cognitive overload/tiredness | Can you walk me through the process that is used to decide how many vaccines to order?    Can you walk me through the process that is used to decide to whom vaccines will be offered, among the inmates? |
| Environmental context and resources  (Any circumstance of a person's situation or environment that discourages or encourages the development of skills and abilities, independence, social competence, and adaptive behaviour) | Environmental stressors  Resources/material resources  Organizational culture/climate  Salient events/critical incidents  Person-environment interaction  Barriers and facilitators | Can you walk me through the process that is used to decide how many vaccines to order?    Can you walk me through the process that is used to decide to whom vaccines will be offered, among the inmates? |
| Motivation |  | Are staff at the jail required to get the flu/COVID-19 vaccines?  PROBE: If not, how do you think that workers decide whether or not to receive the vaccines?  Now that the COVID-19 vaccine is available to HSAs and populations living in jails, do you have a target percentage of vaccination of inmates? Of people who work at the jail? |
| Optimism  (The confidence that things will happen for the best or that desired goals will be attained) | Optimism  Pessimism  Unrealistic optimism  Identity | What thoughts do you have about the spread of infectious diseases through your institution in the short-term?  In the long-term? |
| Beliefs about consequences (Acceptance of the truth, reality, or validity about outcomes of a behaviour in a given situation) | Beliefs  Outcomes expectancies  Characteristics of outcome expectancies  Anticipated regret  Consequents | How do you feel about increasing the rates of vaccination in your institution?  PROBE: What are some of the benefits/challenges?  PROBE: Think about influenza, for example.    In your experience, what are the attitudes that you’ve seen among the other HSAs around getting vaccinated themselves? (i.e. is it a hassle, does it make them feel safer….?) |
| Goals  (Mental representations of outcomes or end states that an individual wants to achieve) | Goals (distal/proximal)  Goal priority  Goal/target setting  Goals (autonomous / controlled)  Action planning  Implementation intention | How important is it to you to improve vaccination rates at your institution? |
| Behavioral regulation (Anything aimed at managing or changing objectively observed or measured actions) | Self-monitoring  Breaking habit  Action planning | What are some barriers to vaccination of inmates? Of HSAs?  PROBE: other than health concerns, why are some inmates not getting vaccinated?  How is your jail planning to address these barriers? |
| Reinforcement (Increasing the probability of a response by arranging a dependent relationship, or contingency, between the response and a given stimulus) | Rewards (proximal/distal, values/not valued, probable/improbable)  Incentives  Punishment  Consequents  Reinforcement  Contingencies  Sanctions | In your opinion, what potential outcomes of vaccination would lead your institution to promote vaccination for all inmates and/or HSAs (for example: improved morale, lower infection rates, higher compliance of inmates….)    What outcomes might make your colleagues more likely to get vaccinated?    What outcomes might make your inmates more likely to get vaccinated?    How do you feel that offering vaccination affects the way that an inmate interacts with the jail’s medical system? |
| Skills  (an ability or proficiency acquired through practice) | Skills  Skills development  Competence  Ability  Interpersonal skills  Practice  Skill assessment | Can you describe any training that you’ve had in encouraging vaccination among inmates at your institution?    How do you think that your medical training has influenced your personal attitudes towards vaccination?    How do you think that your correctional training has influenced your personal attitudes towards vaccination? |
| Social/Professional role and identity  (a coherent set of behaviors and displated personal qualities of an individual in a social or work setting) | Professional role  Social identity  Identity  Professional boundaries  Professional confidence  Group identity  Leadership  Organizational commitment | Can you describe your personal experience about talking about vaccines to other staff at the jail? With inmates?  PROBE: Have there been times when others who work at the jails have disagreed with you about vaccination?    How do you feel that interactions with others (including your co-workers, friends/family and the inmates with whom you interact) influence your decision to get vaccinated in general?    How might an inmate’s experience(s) with HSA staff impact their willingness to get vaccinated? |
| Beliefs about capabilities  (Acceptance of the truth, reality, or validity about an ability, talent, or facility that a person can put to constructive use) | Self-confidence  Perceived competence  Self-efficacy  Perceived behavioral control  Beliefs  Self esteem  Empowerment  Professional confidence | How do you think that your natural ability to fight off an infectious disease, like the flu, without a vaccine affects your desire to get vaccinated?  PROBE: Tell me more about that… |
| Emotion  (A complex reaction pattern, involving experiential, behavioural, and physiological elements, by which the individual attempts to deal with a personally significant matter or event) | Fear  Anxiety  Affect  Stress  Depression  Positive/negative affect | How do your feelings about vaccination influence your decision to get vaccinated?  PROBE: Which emotion (i.e. hopefulness, fear, anxiety, etc…) most significantly determines your decision to get/not get vaccinated regularly? |

**Note.* The domains and constructs were defined in “Validation of the theoretical domains framework for use in behaviour change and implementation research” by Cane, J., O’Connor, D. & Michie, S.^17^

**Legend:**

- - HSA: Health Services Administrator
  - Flu: Influenza
  - COVID-19: Coronavirus Disease 2019

*Supplement 2. Interview Guide*

**1. Knowledge:**

What types of vaccines do you give in jail?

**PROBE**: are any of these vaccines mandatory?

How do you think your jails does compared to other jails with getting people vaccinated?

**2. Behavioral regulation:**

Tell me about the process of giving someone a vaccine in jail.

**PROBE**: is this process different depending on the type of vaccine (i.e. flu, Hep A, etc)?

How was this process developed?

**PROBE**: Do you know if any guidelines were used to develop this process?

**3 and 4. Memory, attention and decision processes and Environmental context and resources:**

Can you walk me through the process that is used to decide how many vaccines to order?

Can you walk me through the process that is used to decide to whom vaccines will be offered, among the inmates?

**5. Motivation:**

Are staff at the jail required to get the flu/COVID-19 vaccine?

**PROBE:** If not, how do you think that workers decide whether or not to receive

the vaccines?

Now that the COVID-19 vaccine is available to HSAs and populations living in jails, do you have a target percentage for vaccination of inmates? Of people who work at the jail?

**6. Optimism:**

What thoughts do you have about the spread of infectious diseases through your institution in the short-term? In the long-term?

**7. Beliefs about consequences:**

How do you feel about increasing the rates of vaccination in your institution?

**PROBE**: What are some of the benefits/challenges?

**PROBE:** Think about the flu, for example.

In your experience, what are the attitudes that you’ve seen among the other HSAs around getting vaccinated themselves? (i.e. is it a hassle, does it make them feel safer….?)

**8. Goals**

How important is it to you to improve vaccination rates at your institution?

**9. Behavioral regulation:**

What are some barriers to vaccination of inmates? Of HSA workers?

**PROBE**: other than health concerns, why are some inmates not getting

vaccinated?

How is your jail planning to address these barriers?

**10. Reinforcement:**

In your opinion, what potential outcomes of vaccination would lead your institution to promote vaccination for all inmates and/or HSA workers? (for example: improved morale, lower infection rates, higher compliance of inmates….)

What outcomes might make your colleagues more likely to get vaccinated?

What outcomes might make your inmates more likely to get vaccinated?

How do you feel that offering vaccination affects the way that an inmate interacts with the jail’s medical system?

**11. Skills:**

Can you describe any training that you’ve had in encouraging vaccination among inmates at your institution?

How do you think that your medical training has influenced your personal attitudes towards vaccination?

How do you think that your correctional training has influenced your personal attitudes towards vaccination?

**12. Social or Professional role and identity/Social influences**

Can you describe your personal experience about talking about vaccines to other staff at the jail? With inmates?

**PROBE:** Have there been times when others who work at the jails have

disagreed with you about vaccination?

How do you feel that interactions with others (including your co-workers, friends/family and the inmates with whom you interact) influence your decision to get vaccinated in general?

How might an inmate’s experience(s) with HSA staff impact their willingness to get vaccinated?

**13. Beliefs about capabilities:**

How do you think that your natural ability to fight off an infectious disease, like the flu, without a vaccine affects your desire to get vaccinated?

**PROBE**: Tell me more about that…

**14. Emotion:**

How do your feelings about vaccination influence your decision to get vaccinated?

**PROBE:** Which emotion (i.e. hopefulness, fear, anxiety, etc…) most significantly determines your decision to get/not get vaccinated regularly?

**Legend:**

- - HSA: Health Services Administrator
  - Flu: Influenza
  - COVID-19: Coronavirus Disease 2019

*Supplement 3. Transcript Codebook*

| **Code** | **Definition of what it means** |
| --- | --- |
| Beliefs, attitudes about health and prevention | Personal feelings (beliefs and attitudes) regarding vaccination determine individual's willingness to be vaccinated |
| Benchmarking: external comparisons | Compares current vaccination rates to past vaccination rates **in different facilities** |
| Benchmarking: internal comparisons | Compares current vaccination rates to past vaccination rates **in the same facility** |
| Communication/Media | Hearing information (true or not) from others or from the media as an influence on vaccine hesitancy |
| Community | How affairs in the community, outside of the jail, affect vaccination practices in jail |
| Complacency vs. Aspiration | Reference to respondent's level of acceptance of current vaccination rates in their jail and the amount of consideration given to increasing vaccination rates  **Complacency:** OK with current rates  **Aspiration:** respondent intimates that they want higher vaccination rates |
| Costs: Financial | The financial price of a vaccine as a determinant of willingness to get vaccinated |
| Design of the Vaccination Program/ Mode of Delivery | 1) Whether or not (and how) a specific program/methodology was designed to handle offering/ordering/distribution of vaccines in the jail; whether or not interviewee knows how vaccination program was developed  2) How vaccination is offered/promoted methodically in jail  3) requirement among staff to get vaccinated outside of the jail |
| Free Will vs. Obligation | Choosing to get vaccinated (or not) rooted in personal decision-making (free will) or feeling compelled by reasons other than personal choice (obligation) |
| Health Systems and Providers Trust | 1) How the strength of a potential recipient's trust in their provider or their healthcare system acts as a facilitator of vaccination  2) How the vaccination might strengthen the trust a recipient has in their jail's health system/provider |
| Immunization: Social Norm vs. Not Needed | References motivation to get vaccinated as rooted in need to do it for others/societal good or references a rejection of this idea |
| Influential Leaders, Immunization Program Gatekeepers and Anti- or Pro-Vaccination Lobbies | 1) References to the involvement and/or stance taken by these individuals/groups as a facilitator/barrier to promoting vaccination in jails  2) leading by example in jail |
| Introduction of a New Vaccine | Reference to attitudes regarding vaccination being influenced by the novelty of a vaccine |
| Logistics | Logistics (i.e. ease, hassle, number of steps) in the ordering and/or administration vaccinations referenced as a facilitator/barrier |
| Peer Communication | Conversations with peers (i.e. HSA to HSA, inmate to inmate) about vaccination |
| Peer Pressure | Using the opinions of peers to impede or facilitate vaccination in jail |
| Personal, Family and/or Community Members’ Experiences with Vaccination, Including Pain | Reference to past experiences with vaccination (positive and/or negative) |
| Policies/Politics | Internal or external policies and/or politics determine institutional norms/attitudes towards vaccination |
| Reliability/Source of the Vaccine | Recipients/Interviewees’ feelings about the source of the ingredients in the vaccine and/or their thoughts on the reliability (ability to do its job) of the vaccine |
| Religion/Culture/ Gender/SES | How the recipient's religion/culture/gender/ socioeconomic status determines their willingness/hesitancy to be vaccinated |
| Risk/Benefit (Perceived/Heuristic) | Reference to someone weighing the what they personally perceive the risks and benefits of vaccination to be as a factor in their attitude towards vaccination |
| Risk/Benefit (Epidemiological and Scientific Evidence) | Reference to someone weighing the scientifically studied/epidemiological risks and benefits of vaccination as a factor in their attitude towards vaccination |
| The Strength of the Recommendation and/or Knowledge Base and/or Attitude of Healthcare Professionals | Reference to how the intensity of the recommendation/knowledge of healthcare providers as a barrier/facilitator of vaccination |
| Trust in the Healthcare System and Healthcare Providers | Trust in healthcare providers as a motivator/deterrent for vaccination among incarcerated |
| Turnover | Transient nature of incarcerated population as a facilitator or barrier of vaccination initiatives |

**Legend:**

- - HSA: Health Services Administrator
  - COVID-19: Coronavirus Disease 2019

*Supplement 4. Weight of codes applied to interview transcripts and themes emergent from those codes.*

| **Theme** | **Code** | **N of Interviewees Who Referenced** | **N of Total Times Code was Applied** |
| --- | --- | --- | --- |
| **Incarceration as an opportunity for vaccination** | Communication/Media | 3 | 3 |
|  | Community | 3 | 3 |
|  | Costs: Financial | 1 | 1 |
|  | Design of the Vaccination Program/Mode of Delivery | 7 | 20 |
|  | Free Will vs Obligation | 8 | 28 |
|  | Influential Leaders, Immunization Program Gatekeepers and Anti- or Pro-Vaccination Lobbies | 6 | 13 |
|  | Logistics | 7 | 17 |
|  | Peer Pressure | 1 | 1 |
|  | Religion/Culture/Gender/SES | 5 | 6 |
|  | The Strength of the Recommendation and/or Knowledge Base and/or Attitude of Healthcare Professionals | 6 | 11 |
|  | Trust in the Healthcare Systems and Healthcare Providers | 8 | 17 |
|  | Turnover | 5 | 6 |
| **Personal Views on Vaccination/ Personal Choice** | Beliefs, attitudes about health and prevention | 7 | 19 |
|  | Communication/Media | 3 | 3 |
|  | Community | 3 | 3 |
|  | Free Will vs. Obligation | 8 | 28 |
|  | Immunization: Social Norm vs. Not Needed | 6 | 13 |
|  | Influential Leaders, Immunization Program Gatekeepers and Anti- or Pro-Vaccination Lobbies | 6 | 15 |
|  | Introduction of a New Vaccine | 5 | 12 |
|  | Peer Communication | 5 | 10 |
|  | Peer Pressure | 1 | 1 |
|  | Personal, Family and/or Community Members’ Experiences with Vaccination, Including Pain | 5 | 12 |
|  | Policies/Politics | 2 | 2 |
|  | Reliability/Source of the Vaccine | 0 | 0 |
|  | Religion/Culture/ Gender/SES | 5 | 6 |
|  | Risk/Benefit (Perceived/Heuristic) | 7 | 24 |
|  | Risk/Benefit (Epidemiological and Scientific Evidence) | 5 | 19 |
|  | The Strength of the Recommendation and/or Knowledge Base and/or Attitude of Healthcare Professionals | 6 | 11 |
|  | Trust in the Healthcare System and Healthcare Providers | 8 | 14 |
| **Need for Jail Vaccine Protocol Modifications** | Benchmarking: external comparisons | 6 | 7 |
|  | Benchmarking: internal comparisons | 1 | 1 |
|  | Complacency vs. Aspiration | 8 | 39 |
|  | Design of the Vaccination Program/Mode of Delivery | 7 | 20 |
|  | Logistics | 7 | 17 |
|  | Policies/Politics | 2 | 2 |

**Legend:**

- - SES: socioeconomic status
